# An overview of the articles published in the Indian Journal of Pharmacology during the year 2007

**DOI:** 10.4103/0253-7613.45159

**Published:** 2008

**Authors:** D.M. Parmar, S.P. Jadav

**Affiliations:** Department of Pharmacology, M.P. Shah Medical College, Jamnagar-361008, Gujarat, India. E-mail: drdinesh06@rediffmail.com

On the arrival of the sixth issue (December 2007), the Indian Journal of Pharmacology (IJP) had completed its journey for the year 2007 under the new editorial team headed by Dr. Shiv Prakash, the present chief editor of the IJP.

In this issue of the IJP (Indian J Pharmacol 2007;39:303-6), for the first time, the report on the workshop by Pitchai Balakumar *et al*. was published. This preconference workshop on “Basic concepts of scientific research and communication” was held at Chandigarh during the 40^th^ Annual Conference of the Indian Pharmacological Society, 2007.

We read this workshop report with great interest and felt that it is really like a boon for those who were not able to attend this workshop, like us. This prompted us to think that why not write a brief report/summary of the articles published in the corresponding year in the IJP. Usually, the author index as well as the title index is published in the last issue of the IJP for the concerned year. Undoubtedly, while this information serves as an important tool in searching the articles, it could limit the search of articles of one's own interest. Therefore, the attention toward some other important articles would be decreased and/or ignored. Considering this aspect the purpose of writing such a report to create and/or increase interest in large numbers of articles seems justified and hence we have tried to write this article for the benefit of the readers and contributors of the IJP. A total 79 articles have been accommodated in different sections in 312 pages of the 6 issues of the IJP published in the year 2007. We have distributed these articles in suitable categories and mentioned a brief conclusion of at least one article under each category. (Numbers in the parenthesis indicate total number of articles in that category.)

## Renal and cardiovascular function: (7)

Antihypertensive effect of a crude aqueous leaf extract of *Viscum album* (mistletoe) possibly through involvement of sympathetic mechanism (Issue 1, page 15).

## Bone diseases: (2)

*Boswellia serrata* extract was found superior to valdecoxib in terms of safety, efficacy, and duration of action in patients of osteoarthritis of knee (Issue 1, page 7).

## Endocrine function: (1)

Significant postcoital anti-implantation activity of the ethanolic root extract of *Momordica cymbalaria* Fenzl in rats and it may not be due to estrogenic or progestrogenic activities (Issue 2, page 90).

## Antioxidant/free radical scavenging activity: (7)

The ethyl acetate fraction of *Acaria arabica* bark is a free radical scavenger and hepatoprotective (Issue 1, page 33).

## Central nervous system: (5)

Dichloromethane (DcM) fraction of *Kielmeyera coriacea* produced an antidepressant-like effect in the forced swimming test (FST) and interacted with 5-HT_1A_ receptor ligands (Issue 2, page 75).

## Pharmacoepidemiology: (2)

Prescribing pattern of drugs in tertiary care allopathic and ayurvedic hospitals (Issue 1, page 52).

## Cancer/Immunosuppressants: (5)

Lantadene A (LA) induces efficient cell apoptosis by activating the caspase-3 pathway and through downregulation and upregulation of Bcl-2 and Bax expression, respectively (may be developed as a novel chemotherapeutic agent for cancer) (Issue 3, page 140).

## Undergraduate pharmacology education: (2)

Clinical orientation to the MBBS students by demonstrations of various clinical procedures (e.g., setting up the intravenous infusion), demonstration of correct use of the special dosage forms (e.g., use of metered dose inhaler with spacer), and many others (Issue 1, page 57).

## Postgraduate pharmacology education: (2)

Importance of postgraduate education in medical pharmacology – opportunities to make carrier in academia, pharmaceutical industries, and clinical research organizations (Issue 4, page 171).

## Toxicity studies: (5)

Water-based extract of *Labisia pumila* var. *alanta* do not pose any significant reproductive toxicity or complication in pregnancy, delivery, and early pup growth in rats (Issue 1, page 30).

## Gastrointestinal system: (2)

*Euphoria prostrata* possesses bactericidal and antidiarrheic properties and could be a therapeutic alternative for diarrheas of bacterial etiology (Issue 5, page 240).

## Respiratory system: (1)

Mast cell stabilizing property of *Coleus aromaticus* in rat mesenteric tissue (Issue 2, page 119).

## Antimicrobials: (2)

Single preoperative dose of netilmicin is a promising (safe and effective) prophylactic approach in genitourinary surgery (Issue 2, page 121).

## Drug delivery system: (2)

Rectal paracetamol can be used as a safe, effective, and more acceptable analgesic alternative in children (Issue 4, page 187).

## Methods: (6)

Low cost and reproducibility of plexiglass clip-induced hypertension (a favorable opportunity to induce experimental renal hypertension and for preclinical evaluation of new antihypertensives) (Issue 1, page 25).

## Drug interactions: (3)

Famotidine produced less pharmacodynamic drug interaction with pipecuronium as compared to roxatidine in rats (such interaction should be explored in clinical practice) (Issue 1, page 20).

## Therapeutic drug monitoring: (2)

The appropriate use of therapeutic drug monitoring (TDM) may minimize the risk of rejection after transplantation (Issue 2, page 66).

## Pharmacovigilance: (5)

Azathioprine-induced shock in a patient of airborne contact dermatitis (the potentially hazardous and treatable complication of this commonly prescribed drug) (Issue 2, page 115).

## Poisoning: (3)

An educational forum (Issue 2, page 71) and a correspondence (Issue 3, page 170) relating to “Controversies in the management of organophosphate poisoning”.

## Geriatric pharmacology: (1)

Age-related susceptibility to chronic haloperidol-induced orofacial dyskinesia (Aging process leads to increased dopamine sensitivity and increased generation of free radicals which are important causes for initiation of tardive dyskinesia) (Issue 6, page 269).

## Others: (14)

Review articles, viz.; pharmacological aspects and bioavailability enhancement approaches of silymarin (Issue 4, page 172), issues and perspectives of bioequivalence (Issue 5, page 218), and therapeutic alternatives from venoms and toxins (Issue 6, page 260).

To conclude, it is worthwhile to provide such information in this format in place of the standard author index and the title index that is published at the end of the year. Efforts in this direction could also be helpful to draw attention of the readers toward other articles in addition to those of their own interest.

